# Gut Microbiome Modulation of Glutamate Dynamics: Implications for Brain Health and Neurotoxicity

**DOI:** 10.3390/nu16244405

**Published:** 2024-12-22

**Authors:** Benjamin F. Gruenbaum, Kiran S. Merchant, Alexander Zlotnik, Matthew Boyko

**Affiliations:** 1 Department of Anesthesiology and Perioperative Medicine, Mayo Clinic, Jacksonville, FL 32224, USA; kiran.s.merchant@gmail.com; 2 Department of Anesthesiology and Critical Care, Soroka University Medical Center, Ben-Gurion University of the Negev, Beer-Sheva 84101, Israel; alexander.zlotnik.71@gmail.com (A.Z.); matthewboykoresearch@gmail.com (M.B.)

**Keywords:** blood–brain barrier, depression, epilepsy, glutamate, gut microbiome, neurodegeneration, stroke, traumatic brain injury

## Abstract

The gut–brain axis plays an integral role in maintaining overall health, with growing evidence suggesting its impact on the development of various neuropsychiatric disorders, including depression. This review explores the complex relationship between gut microbiota and glutamate (Glu) regulation, highlighting its effect on brain health, particularly in the context of depression following certain neurological insults. We discuss how microbial populations can either facilitate or limit Glu uptake, influencing its bioavailability and predisposing to neuroinflammation and neurotoxicity. Additionally, we examine the role of gut metabolites and their influence on the blood–brain barrier and neurotransmitter systems involved in mood regulation. The therapeutic potential of microbiome-targeted interventions, such as fecal microbiota transplantation, is also highlighted. While much research has explored the role of Glu in major depressive disorders and other neurological diseases, the contribution of gut microbiota in post-neurological depression remains underexplored. Future research should focus on explaining the mechanisms linking the gut microbiota to neuropsychiatric outcomes, particularly in conditions such as post-stroke depression, post-traumatic brain-injury depression, and epilepsy-associated depression. Systematic reviews and human clinical studies are needed to establish causal relationships and assess the efficacy of microbiome-targeted therapies in improving the neuropsychiatric sequalae after neurological insults.

## 1. Introduction

The gut microbiome, which consists of the trillions of microorganisms living in our digestive system, plays a crucial role in many body functions, including brain health [1,2]. Recent research has highlighted a connection between the gut and the brain, known as the “gut-brain axis” [2]. One important aspect of this relationship is how the gut microbiome affects neurotransmitters, especially glutamate (Glu), which is involved in brain signaling and function. Maintaining a proper Glu balance in the brain is essential for healthy neurological function, as disruptions in this balance can contribute to the progression of neurological disorders, particularly in the context of blood–brain barrier dysfunction caused by neurological insult. Although much has been learned about Glu’s role in these diseases, less is known about how the gut microbiome influences Glu and its potential to reduce its harmful effects on the brain.

This review brings together current research on how the gut microbiome affects Glu levels and looks at how modifying the microbiome might offer new treatments for neurological and mental health conditions. This review aims to explore the link between severe blood–brain barrier (BBB) disruption and elevated extracellular Glu levels in the brain, in the context of neurological insults such as stroke, traumatic brain injury (TBI), and epilepsy, each of which may subsequently elevate the risk for neuropsychiatric sequelae, such as depression. We also gather current evidence linking the gut microbiome with Glu regulation, focusing on the absorption and metabolism pathways that may influence BBB integrity and systemic Glu levels, as well as the potential role of microbiome-based interventions, such as diet modulation, probiotics, and fecal microbiota transplantation (FMT) in minimizing neurotoxicity.

## 2. Glutamate: A Non-Essential Amino Acid

Glutamate (Glu) is known as the most abundant non-essential amino acid and excitatory neurotransmitter, being responsible for around 60% of all neurotransmitter activity in the body [3,4,5]. The brain holds large quantities of Glu, though only a small portion of this Glu is typically found in the extracellular fluid (ECF) [4,6]. The highest concentrations are located within nerve terminals [4,6]. Maintaining Glu predominantly within the intracellular space is crucial to prevent saturation of Glu receptors on postsynaptic neurons, ensuring continuous neurotransmission, and to protect against neuronal death caused by excitotoxicity [7]. L-glutamic acid (L-Glu) is involved in various metabolic processes that are essential for brain function, including communication between neurons and central nervous system (CNS) plasticity [8,9]. Activation of N-Methyl d-Aspartate (NMDA) receptors by Glu is also vital for synaptic changes involved in cognition, memory, and learning [10]. In addition to its role as a protein-building amino acid, Glu also plays key roles, including energy production through its exchange with α-ketoglutarate (AKG), an intermediate of the tricarboxylic acid cycle, and ammonia detoxification via astrocytic conversion to glutamine (Gln) [7]. Glu also serves as the precursor to γ-aminobutyric acid (GABA), the main inhibitory neurotransmitter, necessary for balancing excitatory signals and maintaining overall brain stability [7,8].

## 3. Glutamate Regulation

**Figure 1:**
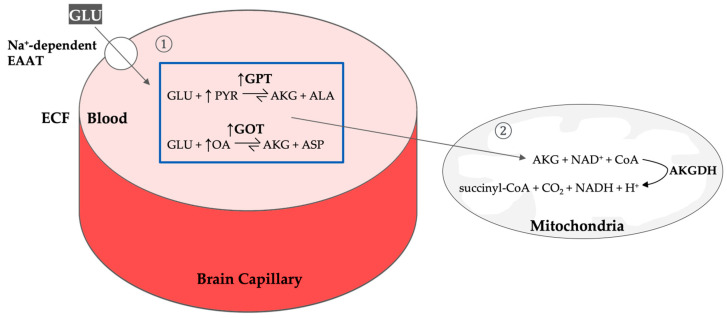
**Brain-to-blood glutamate efflux.** (**1**) In the presence of its enzyme co-substrate pyruvate, GPT catalyzes the reversible conversion of glutamate into its inactive form, 2-ketoglutarate, thereby reducing glutamate levels in the blood. This reduction generates a steep concentration gradient between the extracellular fluid and the blood, enhancing the brain-to-blood glutamate efflux rate. This leads to a reduction in elevated glutamate concentrations in the brain. As long as blood glutamate remains low, this efflux persists. Since the reaction converting glutamate to 2-ketoglutarate is reversible, an accumulation of 2-ketoglutarate can drive the enzyme to regenerate glutamate. (**2**) To sustain glutamate metabolism, 2-ketoglutarate is further degraded by the enzyme 2-ketoglutarate dehydrogenase. By enhancing the concentration gradient between blood and brain glutamate, the brain-to-blood glutamate transport is expedited, thereby mitigating excitotoxicity, associated with elevated brain glutamate levels. AKG, 2-ketoglutarate; AKGH, 2-ketoglutarate dehydrogenase; ALA, alanine; AST, aspartate; CO_2_, carbon dioxide; CoA, Coenzyme A; GLU, glutamate; GOT, glutamate-oxaloacetate transaminase; GPT, glutamate pyruvate transaminase; H^+^, hydrogen ion (proton), NAD^+^, nicotinamide adenine dinucleotide (oxidized form); NADH, nicotinamide adenine dinucleotide (reduced form); OA, oxaloacetate; PYR, pyruvate; succinyl-CoA, succinyl-coenzyme A.

Tight regulation of Glu concentration is essential to balance its critical roles in neurotransmission with its potential for excitotoxic damage [4,11,12]. Recent advances have improved our understanding of how the brain rapidly reduces Glu’s high concentrations (1 mM) in the synaptic cleft to around 1–3 µM, with excess Glu being quickly taken up by sodium (Na^+^)-dependent excitatory amino acid transporters (EAATs) to prevent receptor overstimulation and neurotoxicity [5,12,13]. The brain possesses multiple mechanisms for Glu elimination via brain-to-blood efflux, where excess Glu in the ECF is transported into endothelial cells of brain capillaries and, when concentrations exceed those in the blood, is moved into circulation via EAATs using facilitated diffusion [4,11,14]. The choroid plexus is believed to be involved in this unidirectional brain-to-blood active transport, as observed by Berl et al. [15], who noted rapid clearance of Glu from the CSF to the bloodstream [12,16]. Additionally, Glu in the presence of pyruvate (PYR) and oxaloacetate (OA) is metabolized to AKG in the blood by enzymes such as glutamate-pyruvate transaminase (GPT) and glutamate-oxaloacetate transaminase (GOT) [4,17,18] (see Figure 1). Another pathway involves the Glu-Gln cycle, where astrocytes convert excess Glu into Gln via the endothelial enzyme glutamine synthetase (GS), shuttling excess Gln to neurons for reconversion, maintaining neurotransmitter homeostasis [5,13]. This compartmentalization and efficient clearance quickly reduce Glu to levels where it has neither excessive excitatory nor toxic effects, which supports a healthy, functioning brain [5].

The blood–brain barrier (BBB) plays a crucial role in protecting the brain by limiting the entry of Glu from the bloodstream, a process that is far more restricted compared to other neutral amino acids [18]. Composed of tightly joined endothelial cells, a basement membrane, pericytes, and astrocytic end-feet, the BBB forms a physical barrier that shields the CNS from paracellular flow of harmful substances [8,9,19]. The BBB has multiple layers that control solute flow, requiring blood Glu to penetrate at least five of these layers to enter the brain [9]. Studies by Hutchison et al. [20] have shown that Glu is taken up by isolated brain capillaries through a high-affinity, concentrative transport system, likely present on the abluminal side of the BBB [8]. Under normal conditions, this system allows only a small amount of Glu to cross into the brain, thus maintaining tight regulation within the CNS [9,19].

## 4. Glutamate Toxicity

Lucas and Newhouse [21] were the first to observe the detrimental effects of excess Glu, a ubiquitous neurotransmitter, under physiological conditions, which can become a potent excitotoxin when present in excessive levels, triggering excitotoxicity. This term was coined by John Olney [13] in the 1970s to describe cell death resulting from overstimulation of EAATs [22,23]. Elevated synaptic Glu opens NMDA and amino-3-hydroxy-5-methyl-4-isoaxazolepropionate acid receptors (AMPA), allowing a surge of calcium (Ca^2+^) and Na^+^ ions into neurons [18,21]. This ionic overload leads to oxidative stress, mitochondrial toxicity, cytotoxic edema, and, ultimately, neuronal death [21,24]. While NMDA receptors are central to Ca^2+^ influx, which plays a crucial role in neuronal degeneration, AMPA receptors facilitate Na^+^ entry, adding to the neurotoxic cascade [25,26]. Ionotropic and metabotropic Glu (mGlu) receptors, found on both neurons and glial cells, activate downstream signaling pathways which, under excessive stimulation, can cause widespread neurodegeneration [5,13]. Normally, synaptically released Glu is swiftly cleared by EAATs, keeping its concentration low and preventing chronic receptor activation [4,11,14]. However, in excitotoxic conditions, unregulated Glu levels disrupt cellular homeostasis, contributing to neuronal necrosis and apoptosis [21,23].

Mechanisms to reduce brain glutamate levels have been extensively explored as strategies to limit excitotoxicity and neuronal damage following brain insult (Table 1). Glutamate receptor antagonists, such as NMDA and AMPA receptor blockers, aim to reduce the excessive activation of glutamate receptors that lead to calcium influx and subsequent neuronal injury [27]. Blood glutamate scavenging, which includes therapies such as oxaloacetate administration, works by facilitating the conversion of glutamate to less harmful compounds, effectively lowering extracellular glutamate concentrations [28] (Figure 1). Anticonvulsants, commonly used to manage seizures, can also regulate glutamate release and synaptic activity, thereby minimizing excitotoxicity [29].

In addition to pharmacological approaches, methods such as hemodialysis and peritoneal dialysis have been explored to physically remove glutamate from the systemic circulation [30]. Hormonal therapies, including estrogen and progesterone, have shown neuroprotective effects by modulating glutamate-receptor expression and promoting anti-inflammatory and antioxidative pathways [31]. Collectively, these strategies highlight the multifaceted approaches being investigated to manage glutamate-mediated neurotoxicity and improve outcomes after neural injury.

## 5. Blood–Brain Barrier’s Role in Glutamate Toxicity

Studies indicate that compromised BBB integrity, often due to stress, inflammation, hyperexcitability, or oxidative stress, permits increased permeability, allowing elevated plasma levels of Glu and other substances to enter CNS [19,32]. Additionally, research has shown that chronic stress disrupts brain endothelial cells and the tight junction proteins claudin-5 and occludin, indicating compromised BBB integrity under stressful conditions [9]. In animal models and human studies, this breakdown in BBB function with subsequent rise in brain Glu levels has been associated with numerous acute and chronic neurodegenerative conditions, including stroke, traumatic brain injury (TBI), epilepsy, and even cerebral tumors [9,17,33]. For instance, decreased Glu transport by the glymphatic system has been observed following brain insults [9]. When the BBB’s selective barrier is compromised, brain Glu levels can rise several hundred-fold, following a gradient from plasma to brain, which exacerbates neurotoxicity and neuroinflammation [34].

Studies reveal that even as modest as 10% increases in brain Glu levels above normal may contribute to neurodegenerative conditions such as depression [9,34]. Under normal conditions, EAATs constantly work to maintain Glu homeostasis, but in cases of BBB disruption, these systems are overwhelmed, further linking blood Glu levels to brain Glu concentrations [9]. A study by Boyko et al. [35] further explored this relationship, concluding that the degree of BBB permeability plays a key role in regulating brain Glu levels in both healthy and injured brains [34]. Genetic predispositions, such as polymorphisms in genes encoding tight junction proteins [36], and environmental factors, including chronic diseases, infections, exposure to toxins, and aging [37], can compromise BBB integrity, further amplifying its permeability and disrupting Glu homeostasis [34].

## 6. The Gut–Brain Axis

**Figure 2:**
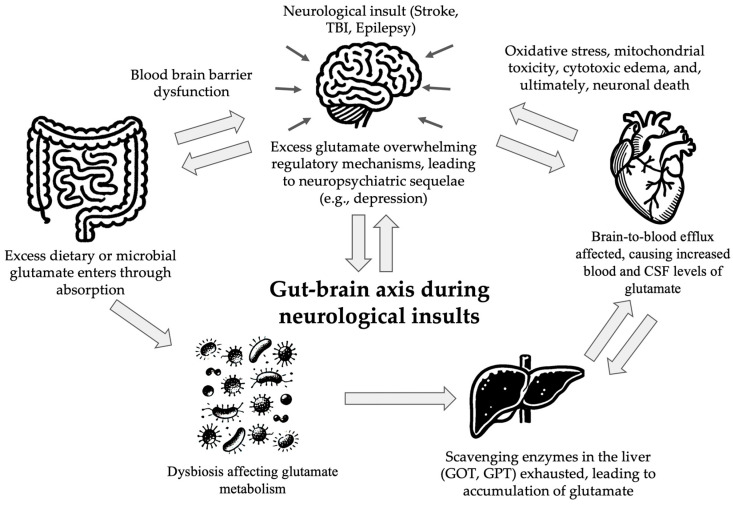
**The disruption of the gut–brain axis and glutamate homeostasis during neurological insults.** Neurological insults overwhelm regulatory mechanisms, leading to excess glutamate in the brain, which contributes to neuropsychiatric sequelae, oxidative stress, mitochondrial toxicity, cytotoxic edema, and neuronal death. These interconnected pathways highlight the role of the gut–brain axis in mediating systemic and neurological effects during insults to the central nervous system. CSF, cerebral spinal fluid; GPT, glutamate pyruvate transaminase; GOT, glutamic oxaloacetic transaminase; TBI, traumatic brain injury.

The relationship between the gut and brain is two-way, involving neural, endocrine, and immune pathways [2] (see Figure 2). The enteric nervous system (ENS), often referred to as the “second brain”, participates in the development of neurological disorders [6,38,39]. The integral role of the gut–brain axis (GBA) is highlighted by the ENS transmitting 100 to 500 million neurons located within the submucosa and myenteric plexi of the intestinal wall, extending from the esophagus to the anus [39]. Structurally and functionally identical to the brain, its regulation, development, and renewal are largely influenced by gut microbiota [39]. Emerging evidence emphasizes the gut microbiome’s influence on distant organs, including the brain [2]. The multitudes of microorganisms colonizing the human gut—including bacteria, viruses, archaea, and fungi—play fundamental roles in wellness and illness [1,2]. Beyond their roles in digestion and immunity, experimental studies further stress the gut microbiota’s role in regulating brain function and behavior through the GBA [40]. For example, a systematic review found an association between antibiotic use and the resulting development of depression, linked to antibiotics’ ability to limit gut microbiota diversity [2].

Microbial dysbiosis refers to a disruption in the balance, composition, and diversity of the gut microbiome, characterized by an excess of microbes producing pro-inflammatory cytokines, and an depletion of microbes responsible for producing anti-inflammatory cytokines [2,39,41]. It increases intestinal permeability due to the downregulation of tight junction proteins such as claudin-5 and occludin, especially in the hippocampus, striatum, and frontal lobe cortex, which leads to “leaky gut syndrome” [41]. This condition can stimulate systemic inflammation and compromise the BBB, promoting the entry of immune cells and bacterial metabolites into the brain [41]. This increases the levels of cytokine and stress hormones in the brain, which may further worsen depressive symptoms [41].

Interestingly, a link between the GBA and Glu has been suggested in relation to the pathogenesis of depression [42]. Glu equalizes quickly across various body fluids and is distributed among organs such as skeletal muscle, the liver, and the gut [17]. Recent studies indicate a potential transmitter role for Glu within the gut, with evidence of EAAT protein expression in enteric neurons [6]. Moreover, research has repeatedly proposed that alterations in gut microbiota can influence brain Glu levels [9]. Consequently, depression has been linked to disruptions in the Glu-Gln-GABA cycles within specific brain regions such as the hippocampus, cerebellum, and striatum [43]. Likewise, studies have revealed a distinct gut microbiota composition in patients compared to healthy controls [2]. Different microbial populations may either promote or inhibit Glu uptake. Similar to how genetic differences affect cholesterol metabolism, variations in microbiome composition could lead to differential regulation of Glu levels across individuals, potentially explaining the variability in susceptibility to Glu-induced neurotoxicity.

Individual variability in Glu absorption may also be influenced by genetic factors that influence microbial composition and functionality. For instance, while some individuals are less affected by dietary Glu and show resistance to excitotoxicity, others may have microbial strains that predispose them to Glu accumulation and increased neurotoxicity. Research is beginning to identify the microbial strains that alter Glu metabolism, which may explain why certain individuals are more vulnerable to neuropsychiatric conditions after neurological insult. Moreover, studies have found specific gut microbiome changes in specific conditions. For example, the microbial composition in PSD rats was significantly distinct, showing disturbances of Lachnospiraceae, Lactobacillaceae, Streptococcaceae, Erysipelotrichaceae, and Ruminococcaceae, with alterations in the gut microbiome closely reflecting behavioral outcomes [44]. In another study, the bacterium Eubacterium ventriosum was shown to be markedly reduced after TBI in mice [45]. In major depressive disorder (MDD), the most affected microbial phyla are Firmicutes, Actinobacteria, and Bacteroidetes, with an increase in Bacteroides and a depletion of Blautia, Faecalibacterium, and Coprococcus [2]. Consistent findings also show an abundance of Eggerthella species and a reduction in Sutterella [2]. These microbial shifts are associated with disruptions in amino acid and lipid metabolism [44].

D-glutamate (D-Glu) is known to be an essential component of the bacterial peptidoglycan cell wall [46]. Several bacterial strains also produce Glu during food fermentation [46]. Lactic acid bacteria, including Lactobacillus plantarum, Lactococcus lactis, and Lactobacillus paracasei, are known for their ability to synthesize Glu [42,46,47]. Furthermore, Glu acts as a precursor for GABA via Glu decarboxylase, which is found in both gram-positive and gram-negative bacteria [46,47]. Non-pathogenic bacteria such as Corynebacterium glutamicum, Brevibacterium lactofermentum, Mycobacterium smegmatis, and Bacillus subtilis can also convert L-Glu to D-Glu [46]. This suggests that the presence of these bacteria in mental disorders may help in better utilization of Glu (resulting in depletion) and increased synthesis of GABA [47].

Additionally, gut microbiota can affect the expression of Glu and GABA receptors in the brain, further reinforcing the gut–brain relationship [41,43]. Clinical and preclinical studies also show that segmented filamentous bacteria may increase susceptibility to depression by encouraging T helper 17 cell production [2]. Additionally, vagus nerve excitation by gut microbiota has been shown to influence the serotonin, GABA, and Glu levels in the brain [45,48]. The gut microbiota also plays a critical role in regulating glial function through signaling pathways [1]. It strongly affects microglial activity, changing them from a proinflammatory to an anti-inflammatory state [1]. When microglia malfunction, it can trigger signals that cause neuroinflammation, which contributes to depression [1,38].

## 7. Glutamate’s Role in Depression

The World Health Organization expects depression to be the leading cause of disease burden in middle- and high-income countries by 2030, with an approximate prevalence of 17% in the US [9]. Depression is a common, coexisting condition associated with TBI, acute ischemic stroke, intracerebral hemorrhage, and chronic neurological disorders, including Parkinson’s disease, Alzheimer’s disease, epilepsy, and multiple sclerosis [9]. Since the first mention of the Glu hypothesis of depression in the 1990s, research has persistently shown a strong association between hyperglutamatergic neurotransmission and numerous psychiatric conditions, especially depression [42,49]. While depressive disorders are typically treated by targeting the serotoninergic, adrenergic, and dopaminergic systems with medications that enhance synaptic availability of these neurotransmitters, this approach proves effective in only about two-thirds of patients [49].

The Glu system is one of the largest neurotransmitter systems in the brain, second only to the GABAergic system. Together, they regulate functions such as sleep/wakefulness, emotional and motivational activities in the neocortex [9]. Changes in glutamatergic excitatory transmission, balanced by inhibitory signals, play a crucial role in cognition and emotion [9]. Studies have found higher Glu levels in the serum, plasma, and CSF of patients with MDD, particularly in the frontal cortex [9]. A study by Mitani et al. [50] found that the plasma Glu levels served as an indicator of disease severity in depression [9]. Moreover, a recent meta-analysis of magnetic resonance spectroscopy has identified disruptions in Glu receptors, such as NMDA, AMPA, and mGlu receptors, in postmortem brain samples from individuals with MDD, reinforcing its role in the disease process [9]. Furthermore, a study of MDD patients showed significantly lower mRNA levels for NMDA and AMPA receptor subunits in the perirhinal cortex, with decreased NMDA receptor subunit levels in the anterior prefrontal cortex [9].

Gln concentrations, a byproduct of Glu conversion, have also been found to be altered in depression, despite normal Glu levels [9]. Alternatively, some evidence suggests that depression results from disruptions in the BBB, which affects Glu efflux [9]. The impact of Glu on depression goes beyond clinical observations. Preclinical studies have shown that diets high in sodium Glu can induce depressive behaviors in rodents, including anhedonia, decreased social interaction, and behavioral despair [9,48]. Given the complex interplay between Glu, BBB integrity, and mood disorders, this growing understanding highlights the potential of focusing on glutamatergic pathways to treat depression [48].

## 8. Neurological Conditions Associated with Depression

### 8.1. Traumatic Brain Injury

Statistics reveal that TBI is a leading cause of mortality and long-term disability across the globe, affecting at least 69 million people annually [33,51]. Following TBI, brain Glu levels exhibit a biphasic spike: an initial surge occurring immediately after injury, followed by sustained elevation persisting for extended periods, sometimes lasting months or even years [11]. The traumatic impact from TBI leads to a loss of astrocytes and neurons, which in turn reduces the availability of EAATs, worsening the buildup of extracellular Glu [9]. While in vitro studies on acute brain slices have recorded extracellular Glu levels ranging from 25 to 90 nM, most in vivo studies using microdialysis have found significantly higher increases, with brain Glu levels between 0.2 μM and 20 μM [9]. The first phase peaks around 5 h post-injury, while the second phase reaches its peak on day 3 [34]. Recent findings indicate that full BBB restoration can take several years following injury [34].

Studies have shown a strong correlation between lower blood Glu levels and improved neurological outcomes after TBI and stroke, highlighting the importance of maintaining Glu homeostasis and avoiding secondary brain injury through cell swelling, apoptosis, and neuronal death [33,49]. Mechanisms contributing to elevated brain Glu in TBI include neuronal death, inflammation, impaired Glu recycling, astrocytic release of adenosine triphosphate (ATP), and BBB destruction [34]. TBI often disrupts the BBB, impairing its role in regulating Glu levels by restricting its clearance [9,35]. Recent research, including Peethambaran et al. [52]’s in vitro model of blast-induced TBI, indicates that membrane integrity changes may further exacerbate secondary injury, underscoring the critical role of BBB integrity in moderating long-term outcomes after TBI [35]. 

TBI is commonly linked to long-term neuropsychiatric issues, including depression, anxiety, and aggression, which can last for decades after the initial injury [34,45]. While these symptoms were once thought to be associated primarily with the emotional burden of physical disability, it is now understood that conditions such as memory impairment, social withdrawal and mood disturbance can occur regardless of injury severity and pain [34]. Research shows that 25% to 50% of individuals experience major depression within the first year after enduring a TBI [9]. Post-TBI depression has proven difficult to treat according to studies such as by Fann et al. [53] and Kreitzer et al. [54], who reported antidepressants to be no more effective than a placebo [49].

Recent research suggests that glutamatergic disturbances, especially a modest but prolonged increase in Glu levels due to BBB damage, contribute to the development of depression after TBI [9,34,48]. Glu diffusion can lead to compromised synaptic integrity and neuronal dysfunction, resulting in behavioral changes such as anhedonia and psychomotor slowing [34]. Additionally, chronic neuroinflammation and BBB breakdown further exacerbate depression by impairing cerebral perfusion and causing brain damage [48]. A large meta-analysis of 392,834 patients reveales that mild TBI significantly increases the chances of developing depression, with an average of 35% [55]. Thus, these neurodegenerative processes ultimately lead to persistent neuropsychiatric symptoms and delays in rehabilitation and recovery [34].

### 8.2. Stroke

Stroke, the third leading cause of mortality globally, often results in long-term disability among survivors [9]. Ischemic stroke, the most common form, leads to restricted blood flow, BBB permeability, and excitotoxicity-induced neuronal death [9]. A hallmark of stroke is the three- to four-hundred-fold elevation of ECF and CSF Glu levels, which trigger intracellular Ca^2+^ overload and worsen neuronal damage beyond the infarcted area and associate strongly with poor neurological outcomes [14,16,21]. While Glu is essential for proper neuronal function, its excitatory properties become harmful to neurons when its concentration in the ECF increases under pathological conditions [21]. Stroke is often associated with cerebral edema, which contributes to secondary brain injury by increasing intracranial pressure [14]. This elevation disrupts cerebral perfusion pressure, leading to reduced oxygen delivery and ultimately causing neuronal necrosis and apoptosis [14]. Therefore, controlling brain edema is crucial for improving outcomes [14]. Notably, a perfect correlation (R = 1.0) has been observed between blood Glu levels and brain edema, highlighting a strong and direct relationship [14].

Elevated Glu levels have also been linked to larger ischemic lesions and worse prognoses in stroke patients [14,16,21]. Under stressful conditions, EAATs can reverse their function, exporting Glu instead of importing it, which contributes to excitotoxic neuronal death [5,6]. Strokes can also cause energy failure, depleting ATP and impairing normal ionic gradients across membranes, leading to Na^+^ and Ca^2+^ influx, which affects multiple cellular compartments, such as cytosol, endoplasmic reticulum, nucleus, and mitochondria [13,40]. This activity, particularly involving AMPA-kainate and NMDA receptors, can severely damage cells [40]. The loss of glial Glu transport causes prolonged increase in plasma Glu levels for up to 24 h after a stroke, suggesting ongoing neuronal damage [13,24]. Studies have shown that lowering Glu levels in the blood improves neurological outcomes in both animal and human models, emphasizing the importance of directing Glu homeostasis in attenuating stroke-related damage [17,33].

According to Canadian Stroke Best Practices [56], all stroke survivors face an increased risk of developing post-stroke depression (PSD), a prevalent mood disorder which often indicates a poor prognosis and elevated mortality risk [40,44]. Approximately one-third of stroke survivors develop PSD within five years, with symptoms typically developing three months post-stroke, and often correlated with lesion size and location [26,40,44]. Cerebral ischemia, caused by reduced or absent blood flow to the brain, triggers ATP depletion, oxidative stress, and neuroinflammation [57]. These events lead to excessive Glu release from intracellular to extracellular spaces, overstimulating NMDA receptors and raising intracellular Ca^2+^ levels, which worsens neuronal injury [57]. Studies have shown significantly elevated Glu and glycine levels in the plasma and CSF of patients with extensive neurological damage, correlating with larger infarct volumes and more severe outcomes [26]. Microdialysis studies, such as by Bullock et al. [58], have observed increased concentrations of these neurotransmitters in these patients, while Kanthan et al. [59] has reported Glu levels 100 times higher than baseline shortly after ischemia [26].

The pathogenesis of PSD involves psychosocial distress, monoamine neurotransmitter alterations, hypothalamic–pituitary–adrenal axis activation, and disrupted energy metabolism [40,44]. Large lesions in critical brain regions such as the left frontal lobe and basal ganglia disrupt mood regulatory pathways, while severe strokes elevate inflammatory cytokines that suppress neurogenic factors, exacerbating depressive symptoms [40]. Dysregulation in Glu metabolism leads to high concentrations, causing neurotoxicity and neuronal loss [40]. This, coupled with a damaged EAAT system, prevents efficient clearance from synaptic clefts, which contributes to mitochondrial dysfunction and impaired sensory transmission [40]. This collectively disrupts mood and emotional regulation, amplifying the severity of PSD [40].

Affecting 20–60% of stroke patients, PSD hinders recovery and quality of life yet remains frequently untreated [40]. Conroy et al. [60] suggested that treating depression in patients with neurological disorders is often more difficult than addressing depression in the general population [40]. Even then, the early initiation of antidepressant therapy has been shown to improve outcomes [9]. Additionally, recent research indicates that stroke survivors have higher suicide rates compared to the population at large [40]. Notably, elevated plasma Glu can serve as a biomarker for PSD risk, emphasizing the need for targeted interventions to address this debilitating complication of cerebral stroke [40].

### 8.3. Epilepsy

Epilepsy, affecting approximately 50 million people worldwide, is characterized by unprovoked seizures ranging from brief loss of attention to prolonged convulsions and loss of consciousness, as seen in status epilepticus (SE) [19]. Glu has been a key focus in epilepsy research due to its significant role in seizure activity [19,61]. Intense seizure activity leads to intracellular Ca^2+^ influx, mitochondrial dysfunction, superoxide production, and caspase activation, resulting in neuronal death [5]. Seizure onset triggers hypersynchronous Glu release, leading to increased systemic Glu levels through diffusion from the ECF to the blood [5,18]. Interictally, extracellular Glu is elevated five-fold in the epileptogenic regions of the human hippocampus, as measured in vivo by Cavus et al. [62], with levels rising six-fold during seizures and remaining elevated for at least 20 min post-seizure [61,63,64].

Seizure activity is also linked to impaired Glu clearance due to deficiencies in enzymes such as GS and disruptions in the glutamate–glutamine cycle [63,64]. Astrocytes play a critical role in this, converting glucose-derived Glu into Gln via GS, yet under pathological conditions such as epilepsy, they become sources of Glu release, aggravating excitotoxicity [63]. Astrocytes also release Glu in response to Ca^2+^ oscillations mediated by AMPA, kainate, and mGlu receptors [63]. Mechanisms include Ca^2+^-dependent exocytosis and astrocytic swelling, which contributes to sudden depolarization events and prolongs seizure duration, although it may not initiate seizures, as reported in studies by Tian et al. [65] and Fellin et al. [61,63,66]. However, some studies suggest that elevated Glu levels may be linked to the initiation of seizures, particularly within the limbic system [35]. Moreover, in cases of GS deficiency, Glu-to-Gln conversion slows down, leading to Glu accumulation, impaired GABAergic neurotransmission, and disrupted excitatory–inhibitory balance [63,64]. Chronic Glu accumulation, worsened by reduced EAATs and enhanced receptor expression (e.g., NMDA receptors), promotes seizure recurrence [19,23].

Additionally, BBB dysfunction in epilepsy exacerbates Glu dysregulation [19]. In turn, excessive Glu release can damage the BBB even further, via endothelial dysfunction and extracellular matrix (ECM) breakdown [19]. Acute BBB disruptions during intense ictal episodes, such as those observed in SE, increase permeability, while chronic BBB damage after recurrent cerebral insults impairs Glu clearance and exacerbates neurotoxicity [19]. Prolonged hyperexcitability also causes GABA receptor internalization and further diminishes its availability [19]. This interplay between astrocytes, neurons, enzymes, and transporters highlights Glu’s role in disrupting the brain’s ability to regulate neuronal firing, and its multifaceted contribution in epileptogenesis [19].

Seizures are the distinguishing features of epilepsy, but emerging evidence highlights a complex interconnection between epilepsy and neuropsychiatric disorders [19]. Patients with epilepsy face a greatly increased risk of developing conditions such as depression and anxiety compared to the general population, with mood and anxiety disorders displaying a reciprocal relationship with epilepsy [19]. Approximately 30% of epilepsy patients experience depression and anxiety, markedly impacting their quality of life and treatment outcomes [19]. Growing research suggests that shared pathophysiological mechanisms, including altered neurotransmitter release, neuroinflammation, and structural brain changes, underlie these comorbidities [19].

Glu plays an integral role in learning, memory, depression, and anxiety [19]. Glu-driven insults, including elevated concentrations during post-ictal periods, intensify Glu-associated neurotoxicity, amplifying neuropsychiatric symptoms in epilepsy [19]. Glu-driven inflammation has been linked to mood disorders, with increased neuroinflammation observed in individuals with depression [19]. This inflammation can alter Glu metabolism, adding to symptoms such as anhedonia and psychomotor slowing [19]. Genetic factors further complicate this relationship [19]. Mutations in genes such as matrix metalloproteinase-9 degrade ECM components and disrupt the BBB during epileptic events, increasing neuroinflammation and neuronal injury [19]. Similarly, mutations in Glu receptor genes affect NMDA receptor signaling, promoting hyperexcitability and excitotoxicity, which contribute to both seizure activity and neuropsychiatric disorders, including depression [19].

Additionally, genetic alterations in Glu metabolism, such as variations in the EAATs, compromise synaptic Glu clearance, resulting in elevated ECF Glu levels [19]. This hyperglutamatergic state promotes both seizure progression and the neurodegenerative processes underlying depression [19]. Together, these interdependent mechanisms emphasize the complex interplay between epilepsy, Glu dysregulation, and neuropsychiatric comorbidities [19].

## 9. Glutamate Metabolism and the Liver

Beyond the intestines, the liver plays a central role in Glu metabolism, converting Glu to Gln via ammonia detoxification pathways [6]. This process prevents Glu accumulation and maintains the balance necessary for CNS wellbeing. Research has revealed that the liver, gut, and skeletal muscles are the primary redistribution sites for the majority of the circulating Glu [67]. Liver-derived scavenging enzymes in the plasma, GOT and GPT, in the presence of their co-substrates, OA and PYR, convert Glu into AKG, aspartate, and alanine [14,33,67].

In preclinical studies, GOT is released into the blood during some disease states, and has been shown to efficiently remove Glu from the brain through its degradation, reducing ischemic damage by about 80% [16,21]. This suggests an innovative neuroprotective approach against ischemic insult [16]. Furthermore, OA has been shown to significantly improve both short- and long-term neurological outcomes after moderate to severe TBI, reduce brain edema, and improve neuronal survival in different hippocampal regions [28]. Similarly, PYR has demonstrated beneficial effects in both TBI and stroke, including improved neurological outcomes, reduced mortality, decreased BBB permeability, smaller brain lesions on histological examination, and increased neuronal survival [11,17]. The neuroprotective effects of OA and PYR may involve multiple mechanisms, such as activating PYR dehydrogenase to restore ATP levels in the cells, and functioning as direct antioxidants [28].

Findings by Campos et al. [16] showed that patients with poorer outcomes exhibited higher blood Glu levels and lower GOT levels at admission [16,18,33]. Conversely, elevated GOT levels at admission were independently linked to better functional outcomes at three months, as well as reduced lesion volume [16,17]. An independent cohort of stroke patients supported this direct correlation, where higher blood Glu levels were linked to worse outcomes and vice versa [17,33,34]. Additionally, a previous study by Campos et al. [68] demonstrated that activation of GOT reduced blood and cerebral Glu levels, leading to a decrease in infarct size, reduced edema volume, and improved sensorimotor outcomes [16,17].

The liver’s critical role in Glu metabolism becomes apparent during chronic liver disease (CLD), where dysregulation in Glu metabolism, either from liver dysfunction or increased systemic ammonia, can disrupt Glu-Gln balance, contributing to neurotoxicity and BBB permeability changes [6]. Ammonia, a byproduct of protein metabolism, is an important substrate for Gln synthesis in the liver. Overtime, when ammonia is not adequately converted due to compromised liver function, a reduction in Glu uptake can contribute to its toxicity [6]. The delayed impact of these effects has been recognized for nearly 100 years; for example, cortical tissue necrosis resulting from focal trauma can increase dramatically (up to 150% in volume) within a day due to secondary effects such as inflammation and the accumulation of ammonia [6]. This highlights how CLD not only disrupts Glu metabolism but also triggers a series of short- and long-term consequences, emphasizing liver’s importance in maintaining metabolic balance. Hence, given Glu’s pivotal role in the neurological trauma, this neurotransmitter appears as a promising target for developing neuroprotective strategies in ischemic stroke [16,21].

## 10. Gut Microbiome Modulation and Diet’s Influence on Glutamate Levels

Gut microbiome modulation and dietary interventions have been shown to influence glutamate levels, offering additional potential therapeutic avenues for conditions characterized by glutamate dysregulation and excitotoxicity (Table 1).

**Table 1:** Potential therapeutic approaches specific to the gut–brain axis for reducing neurotoxic glutamate levels after neurological insults.

Strategies	Mechanism of Action	Potential Clinical Application
Glutamate receptor antagonists (NMDA, AMPA)	Block excessive glutamate signaling, enhancing neuroprotection [27].	Treatment of neurodegenerative disorders such as Alzheimer’s and Huntington’s disease.
Blood–brain barrier protection	Improving BBB integrity to limit glutamate entry into CNS, reducing neurotoxicity [34].	Prevention of excitotoxicity in neurological disorders such as stroke and multiple sclerosis.
Blood glutamate scavengers: Enzymes (e.g., GOT, GPT)	Enzymes metabolize glutamate into non-toxic compounds [28].	Potential therapy for stroke and traumatic brain injury.
Blood glutamate scavengers: Pyruvate, Oxaloacetate	Scavenges free radicals and enhances glutamate metabolism through pyruvate dehydrogenase activation [28].	Therapy for acute neurological insults such as stroke and traumatic brain injury.
Blood glutamate scavengers: Enhanced glutamate breakdown (e.g., Alpha-Ketoglutarate, Alanine, Aspartate Supplementation)	Promotes metabolic conversion of excess glutamate into intermediates that feed into the tricarboxylic acid (TCA) cycle by promoting continuous glutamate metabolism, reducing glutamate toxicity [9,69].	Management of glutamate-related disorders, including epilepsy, neurodegeneration, or mood disorders.
Anticonvulsants (e.g., Valproate, Lamotrigine)	Inhibit glutamate release by regulating Na^+^ and Ca^2+^ channels [29].	Treatment of epilepsy, traumatic brain injury, and seizure disorders.
Hemodialysis and peritoneal dialysis	Facilitates the clearance of neurotoxic metabolites and pro-inflammatory molecules that cross the gut–brain axis, potentially reducing systemic inflammation and BBB disruption [30].	Adjunct therapy for conditions involving neuroinflammation, such as depression or cognitive decline.
Estrogen and Progesterone Therapy	Modulates gut microbiota composition, reduces neuroinflammation, and promotes BBB integrity through hormonal signaling pathways [31].	Treatment of neuropsychiatric disorders influenced by gut–brain interactions, such as postpartum depression or menopausal-related mood changes.
Gut microbiome modulation: Ketogenic diet	Increased fatty acids increase mitochondrial uncoupling proteins, reducing glutamate transamination to excitatory aspartate which reduces seizures and improves neuronal function [70].	Dietary intervention for epilepsy, traumatic brain injury, and depression.
Gut microbiome modulation: Monosodium glutamate reduction	Limits dietary glutamate absorption, and controls plasma and brain glutamate levels [71].	Dietary modification for managing glutamate-induced toxicity.
Gut microbiome modulation: Probiotics	Strains, such as Lactobacillus and Bifidobacterium, modulate gut microbiota and influence glutamate metabolism [72].	Potential therapeutic option for mood disorders such as depression.
Gut microbiome modulation: Fecal microbiota transplants	Alter microbiome composition to regulate gut-derived glutamate absorption and reduce neuroinflammation [73].	Emerging therapy for depression, Parkinsonism, and post-stroke cognitive impairment.

### 10.1. Ketogenic Diet

Research highlights the significant impact of diet on Glu regulation, which, in turn, affects neurological outcomes [48]. Despite this, there remains a lack of comprehensive data on how luminal Glu, whether coming from dietary or microbial sources, might contribute to depression pathophysiology [42]. Dietary interventions, such as the ketogenic diet, have shown promise, with over 50% of patients experiencing a reduction in seizure frequency and many maintaining prolonged remission [74]. This effect may stem from increased fatty acid levels, which increase mitochondrial uncoupling proteins, providing protection against conditions such as Glu toxicity, TBI, and Parkinsonism [74]. Ketosis induced by a ketogenic diet can alter metabolism of neuroactive compounds, including Glu and GABA [74]. Specifically, ketosis increases astrocytic production of Gln and may reduce the transamination of Glu to aspartate, favoring its decarboxylation to GABA, adding to the diet’s antiepileptic effects [74].

### 10.2. Monosodium Glutamate Reduction

Studies on monosodium Glu (MSG) further highlight the relationship between dietary Glu and neurological effects [67]. Using microdialysis, researchers have seen a notable increase in brain ECF Glu levels after large systemic doses of MSG [32]. Rutten et al. [75] observed that oral Glu administration caused a peak in plasma Glu levels after approximately 80 min on average [48]. Subsequent doses did not significantly increase levels further, indicating a saturation point in intestinal absorption and clearance [48]. Moreover, high doses of MSG in neonatal rats have shown to induce overactivation of Glu receptors, causing amino acid irregularities and seizures [48]. In adult rats, MSG administration reduced hippocampal cyclic-AMPK levels and doubled Fas ligand expression, a marker of cell death [48]. All these findings suggest that diets high in MSG may increase blood Glu levels, potentially leading to hyperglutamatergic neurotransmission [48]. Human studies indicate that meals rich in MSG increase circulating blood and muscle Glu levels within hours of consumption [67]. Stegink et al. [76] reported that carbohydrate-rich meals increase intestinal Glu absorption, while MSG-free meals did not affect plasma Glu levels [67]. Other research suggests that dietary Glu has a minimal effect on blood levels due to its rapid metabolism in humans [67]. Given these insights, the dietary regulation of Glu may act as a potential strategy for preventing neurodegenerative processes and treating depression. A Glu-poor diet could help control Glu levels, reducing the risk of hyperglutamatergic transmission and associated neurological disorders [48].

### 10.3. Probiotics

Probiotics, particularly strains from the *Lactobacillus* and *Bifidobacterium* genera, influence glutamate metabolism by expressing glutamate decarboxylase (GAD), enabling the enzymatic conversion of Glu to GABA [72,77]. This conversion is significant in reducing extracellular glutamate concentrations, thereby mitigating the risk of excitotoxicity. In addition to direct metabolic effects, probiotics influence glutamate homeostasis by modulating neuroinflammation, a critical factor in glutamate dysregulation [78]. Probiotics have been shown to reduce the production of pro-inflammatory cytokines and enhance the gut barrier integrity, collectively attenuating systemic inflammation and its adverse effects on the central nervous system [79]. Neuroinflammatory states are associated with impaired glutamate uptake and metabolism, contributing to its accumulation and excitotoxic effects [80]. By addressing these inflammatory processes, probiotics may support neuroprotection by restoring the balance between excitatory and inhibitory neurotransmitters [81]. These findings suggest a therapeutic potential for probiotics in managing glutamate-mediated neuropathologies, including Alzheimer’s disease [82], Parkinson’s disease [83], stroke [84], TBI [85], MDD [38], and seizures [86,87]. Further research is warranted to delineate the specific strains, mechanisms, and clinical applications of probiotics in this context.

### 10.4. Fecal Microbiota Transplantation

The end goal is to develop microbiota-based interventions for neurological sequelae. Accumulating evidence links gut microbiota, Glu pathways, and neurological conditions, promoting the microbiota–GBA as a promising target for therapeutic interventions [1]. Fecal microbiota transplantation (FMT), in which fecal flora is transplanted from healthy donors to patients, offers a quick way to restore gut microbiota and has shown promise as an intervention for chronic diseases associated with dysbiosis, especially those affecting brain health [2,88], as summarized in Table 2. FMT may regulate Glu levels by promoting microbial populations that promote Glu breakdown or inhibit its absorption. Such an approach could help reduce neurotoxic exposure within the CNS. It has improved depressive symptoms in preclinical and clinical studies by correcting microbiota composition, reducing neuroinflammation, and repairing intestinal barrier integrity [2]. A study conducted by Xie et al. [88] revealed a significant increase in N-Acetyl-L-glutamate, gamma-L-Glutamyl-L-glutamic acid, and Glycerophosphocholine levels in stool following FMT. Kelly et al. [89] have also demonstrated that transferring the gut microbiota from depressed human patients to germ-free rats induced behavioral and other characteristic features of depression, suggesting a causal role for microbial dysbiosis in depression [2,45]. Moreover, a recent analysis in a mouse model induced by FMT from depressed patients identified major protein alterations in the serum, prefrontal cortex, cecum, and liver [1].

One randomized controlled trial evaluated oral frozen FMT capsules as an adjunct therapy for MDD and found great improvements in depressive symptoms four weeks post-transplantation [2]. The interconnection between gut microbiota, Glu metabolism, and depression sheds light on the reciprocal communication of the microbiota-GBA [38]. This approach is gaining momentum in both research and clinical settings. However, only a limited number of studies have methodically analyzed the connection between the gut microbiome and depression in humans. Furthermore, these studies usually have small sample sizes, failing to establish strong and reliable connections. Further prospective studies are needed to explore the complex relationships between dysbiosis, Glu, and depression and to develop microbiota-based treatments for depressive sequelae following neurological disorders.

## 11. Future Directions and Conclusions

Future research should focus on investigating microbiome-targeted therapies, such as FMT, in clinical settings. Longitudinal studies are critical to assess the effect of gut microbiome regulation on neuropsychiatric sequelae following neurological insults, possibly helping to explore causative relationships. The complex interplay between the gut microbiome, Glu, and neurotoxicity offers an important pathway for understanding and potentially reducing neuropsychiatric consequences. Through mechanisms such as Glu absorption, liver metabolism, and the BBB integrity, the gut microbiome emerges as a key regulator of brain health. Interventions aimed at modulating the gut microbiota show promise as therapeutic strategies for addressing Glu neurotoxicity and its role in neuropsychiatric conditions.

While much research has been conducted on Glu’s role in MDD and other neurological diseases, the study of post-neurological depression (such as PSD, post-TBI, and epilepsy-associated depression) remains limited. We hypothesize that Glu plays a significant role in these sequelae and targeting this pathway could lead to strategies for preventing and treating these debilitating disorders, thereby reducing the burden of these diseases and improving quality of life after major neurological insults. This review has focused on three conditions, but future studies should explore additional avenues where Glu’s role can be applied to reduce depression incidence after other neurological disorders. More prospective trials and studies are needed to validate these relationships and translate them into treatment strategies. Further investigation into these pathways is essential to clarify the specific mechanisms involved, ultimately paving the way for human clinical studies and practical therapeutic applications.

## Figures and Tables

**Table 2:** **Summary of studies exploring the effects of fecal microbiota transplantation (FMT) on neurological conditions in animal and human models**. Key findings highlight the impact of FMT on behavior, neurological function, gut microbiota composition, and related biochemical/metabolic pathways. ABX, antibiotic clearance; ADHD, attention deficit hyperactivity disorder; BBB, blood–brain barrier; CUMS, chronic unpredictable mild stress; EEG, electroencephalogram; ETH, ethanol-treated donors; FMT, fecal microbiota transplantation; GABA, gamma-aminobutyric acid; GE, gut environment; H&E, hematoxylin and eosin (staining); H_2_S, hydrogen sulfide; IBS: irritable bowel syndrome; IBS-SSS, IBS symptom severity score; IHC, immunohistochemistry; MCAO: middle cerebral artery occlusion; MDD, major depressive disorder; PCR, polymerase chain reaction; PSD: post-stroke depression; RCT, randomized controlled trail; SCFA; short-chain fatty acids; TMAO, trimethylamine N-oxide; TNF-alpha, tumor necrosis factor-alpha; WAG/Rij, Wistar Albino Glaxo rats of Rijswijk.

Study Type	Neurological Condition	Key Findings	Model	FMT Delivery Method	Gut Microbiota Effects	Outcome Metrics	Number of Participants/Subjects	Reference
Animal model	Absence epilepsy	FMT partially restored intestinal morphology, modified gut microbiota composition, reduced seizure frequency and duration	WAG/Rij rats (genetic model for absence seizures)	Oral gavage	Decreased Bacteroidetes (4–8 months) and increased Firmicutes WAG/Rig at 4 months: - Bacteroidetes: lower S24-7, higher Odoribacter and Rikenellaceae - Firmicutes: higher Clostridiales, Clostridiaceae, Lachnospiraceae; lower Lactobacillus and Phascolarctobacterium	Seizure frequency and duration, gut morphology changes	98 rats (WAG/Rig and Winstar): - 36 rats (6 groups of 9; for intestinal motility: *n* = 4/group) - 6 month olds (*n* = 50): - WAG/Rig: EEG control (*n* = 5), fecal donors (*n* = 6), antibiotics (*n* = 5), ETH-treated donors (*n* = 6 × 2) - Winstar: Fecal donors (*n* = 6), antibiotics (*n* = 5), antibiotics + FMT (*n* = 6)	Citraro et al. (2021) [90]
Animal model	Depression	Improved mood and behavior, with mechanisms involving neural, inflammatory, endocrine pathways	Specific pathogen-free Sprague-Dawley rats with chronic unpredictable mild stress (CUMS) model	Oral gavage	CUMS group: Increased Bacteroidetes, Proteobacteria, Firmicutes, and Prevotella Post-FMT: Reversed the CUMS-induced changes in Firmicutes and Prevotella abundance; increase in SCFA-producing taxa	Behavioral scores, neurochemical levels	30 rats randomly divided into: Control, CUMS, and CUMS + FMT	Cai et al. (2022) [91]
Animal model	Drug-resistant epilepsy with ADHD/anxiety symptoms	Improvement in ADHD, anxiety-like behavior, and quality of life; increased GABA/glutamate ratio, no significant change in seizure duration pr frequency	Dogs with drug-resistant epilepsy	Rectal gavage	Decreased Firmicutes and a Blautia_A species, increased Ruminococcus species	Behavioral scores, anxiety scores, GABA/glutamate ratio, fecal sample analysis	9 dog (recipients); 1 Australian Shepherd with well-controlled epilepsy and no behavioral problems (donor)	Watanangura et al. (2024) [92]
Animal model	Traumatic brain injury	Improved cognitive function via modulation of microbiome and BBB integrity	Models of gut microbiota dysbiosis in Sprague-Dawley rats	Oral gavage	Increased Clostridium_T and Allobaculum. Enriched genera in amino acid, nucleoside/nucleotide, and fatty acid biosynthesis. Predominant phyla: Firmicutes, Verrucomicrobiota, and Actinobacteriota	Open field, H&E, TEM analysis, IHC staining, western blot, PCR, metabolic analysis	40 rats, divided into 5 groups (*n* = 8/group): Control, GE model, GE+FMT, antibiotic clearance + GE (ABX), antibiotic removal + GE + FMT	Dong et al. (2024) [93]
Animal model	Middle cerebral artery occlusion (MCAO), post-stroke depression (PSD)	Neurological function and depressive symptoms improved; TMAO/H_2_S metabolites decreased	Rats with MCAO and PSD models	FMT: rectal gavage Probiotic: oral gavage Antibiotic: oral gavage Fluoxetine: oral gavage	Reduced TMAO/H_2_S (a metabolite of intestinal flora, associated with neurological function and depressive symptoms in PSD)	Neurological function, forced swim test, metabolite analysis	Part 1: 45 rats, divided into 3 groups (*n* = 15/group): control, MCAO, PSD. Part 2: 54 rats, divided into 6 groups (*n* = 9/group): control, PSD, FMT, antibiotic, probiotic, fluoxetine (positive control)	Li et al. (2024) [94]
Human model	Major depressive disorder (MDD)	No serious adverse events. 12/15 participants found edema tolerable and would repeat treatment. Exploratory data: FMT may improve gastrointestinal symptoms and quality of life.	Adults with moderate to severe MDD	Enema-delivered FMT	Not assessed	Feasibility, acceptability, and safety of FMT	RCT of *n* = 15: FMT (*n* = 10), placebo (*n* = 5)	Green et al. (2023) [95]
Human model	Depression and anxiety	Improvements in IBS symptoms, reductions in anxiety, and depression scores.	IBS patients with diarrhea and anxiety/depression symptoms	Oral FMT capsules	Restored gut balance: Increased bacterial diversity, improved Bacteroidetes, Ruminococcaceae and Firmicutes; reduced Enterobacteriaceae, Prevotella, Proteobacteria, Actinobacteriota, Bacteroides, and Escherichia–Shigella.	IBS symptom score (IBS-SSS), anxiety and depression scores	RCT of *n* = 18: FMT (*n* = 9), control (*n* = 9)	Guo et al. (2021) [96]
Human donor/rat recipient model	Depression	Alterations in microbiota composition and tryptophan metabolism in depressed patients; germ-free rats exhibited depressive features after FMT from depressed humans.	FMT from depressed humans to a microbiota-depleted antibiotic rat model	Oral gavage	Decreased gut microbiota diversity in depressed patients Increased: Thermoanaerobacteriaceae, Eggerthella, Holdemania, Gelria, Turicibacter, Paraprevotella, Anaerofilum Decreased: Prevotellaceae, Prevotella, Dialister.	Sucrose preference, open field, elevated plus maze, forced swim test; gut microbial composition analysis	28 rats: control (*n* = 15), depressed (*n* = 13)	Kelly et al. (2016) [89]
Human donor/rat recipient model	Depression	Induced depressive-like behaviors in mice	FMT from MDD patients to germ-free Kunming mice	Oral gavage	Gut microbiota altered protein expression across GBA tissues; elevated depression-associated proteins (e.g., TNF-alpha)	Behavioral testing, serum protein analysis	Healthy microbiota recipients (*n* = 20), depression microbiota recipients (*n* = 24)	Liu et al. (2021) [97]
